# Antibunching-like behavior of mesoscopic light

**DOI:** 10.1038/s41598-017-16773-9

**Published:** 2017-12-01

**Authors:** Alessia Allevi, Maria Bondani

**Affiliations:** 1 Department of Science and High Technology, University of Insubria, Via Valleggio 11, 22100 Como, Italy; 2 Institute for Photonics and Nanotechnologies, CNR, Via Valleggio 11, 22100 Como, Italy

## Abstract

We present the implementation of a compact setup for the generation of sub-Poissonian states of light exhibiting the analogous of antibunching behavior in the so-called mesoscopic intensity domain. In the scheme, the idler arm of a pulsed multi-mode twin-beam state is directly measured by a photon-number-resolving detector, whereas the signal arm is divided at a balanced beam splitter, at whose outputs other two photon-number-resolving detectors measure the number of photons. The three detectors measure synchronous with each laser pulse. Due to the nonclassical correlations in the twin beam, when a given value of photons is measured in the idler arm, the conditional states obtained in post processing at the two beam-splitter outputs are nonclassical, showing lower-than-one values of the Fano factor and of the photon autocorrelation coefficient. The possibility to engineer sub-Poissonian states nearly approaching the Fock state with one photon is also addressed.

## Introduction

Antibunching is one of the peculiar traits of quantum systems, evidencing the very definition of “photon” as “light quantum”. For this reason, antibunching experiments^1,2^ are routinely exploited to test the quality of single-photon sources^3,4^. The standard technique is based on a Hanbury Brown-Twiss interferometer^5^, in which the emitted light is divided at a 50/50 beam-splitter (BS) and two single-photon detectors are placed at the two outputs. This scheme is used to evaluate the second-order autocorrelation function *g*_*n*_^(2)^, which is not accessible by direct measurement in the case of a single-photon state. The presence of anti-coincidences (*g*_*n*_^(2)^ < 1) between the two outputs reveals antibunching^6–9^. At higher intensity regimes, such as in the so-called mesoscopic intensity domain (see Sect. “Methods” for more details), the analog of anti-coincidences is given by the existence of anti-correlations between the two BS outputs. These anti-correlations are still a signature of quantumness, as they indicate that the light at the BS input is characterized by sub-Poissonian statistics^10^. Since the mesosocopic intensity regime is the domain in which photon-number-resolving (PNR) detectors operate, it is also possible to have direct access to the *g*_n_^(2)^ autocorrelation function at each BS output. PNR detectors are able to resolve the number of photons in individual pulses up to a maximum value and during the last two decades have been exploited for the reconstruction of the statistical properties of light and the observation of nonclassical features^11–20^. Nevertheless, despite the variety of characterization methods, in the mesoscopic regime no measurements attesting the mesoscopic analog of antibunching have been reported so far in the literature, since the production of sub-Poissonian states of light containing sizeable numbers of photons is not straightforward^21,22^.

In this paper we report on the observation of the antibunching-like effect exhibited by conditional sub-Poissonian states of light in the mesoscopic domain. The experimental scheme is based on the realization of a parametric downconversion (PDC) process in which multi-mode twin beam (TWB) states having sizeable numbers of photons are generated. One of the two parties, say the idler, is directly measured by a PNR detector, whereas the signal is divided at a balanced BS, at whose outputs two PNR detectors are placed. When a given value of photons is measured in the idler arm, the conditional states obtained in post processing at the two BS outputs not only exhibit sub-Poissonian statistics and negativity of the quantity *g*_*n*_^(2)^ − 1, but they are also shot-by-shot anti-correlated in the number of photons. All the correlation functions calculated and presented in the paper are expressed in terms of detected photons and are connected to measurable quantities. They rely on the assumption that for the class of detectors we employed the detection process is described by a Bernoullian distribution^11^. To better characterize our detection scheme we also investigate the effects of a variable transmissivity of the BS on the nonclassical character of the conditional states. Moreover, we show that a double conditioning procedure can be used to achieve pairs of high-fidelity single-photon Fock states.

## Results

### Photon-number correlations

At the single-photon level, the antibunching is usually assessed by the negativity of the quantity *g*_*n*_^(2)^−1, where

1 $${g}_{n}^{\mathrm{(2)}}=\frac{\langle :{n}^{2}\,:\rangle }{{\langle n\rangle }^{2}}=\frac{\langle {n}^{2}\rangle }{{\langle n\rangle }^{2}}-\frac{1}{\langle n\rangle }$$

is the Glauber second-order autocorrelation function for photons, $:\cdot :$ indicates the normal ordering operation, and $\langle \cdot \rangle$ defines the mean value.

In our work, we are interested in the experimental verification of antibunching, thus we express all the quantities connected to the antibunching effect in terms of measurable quantities. We assume the detection operation of PNR detectors be described by a Bernoullian process, so that the link between the statistical properties of photons and those of detected photons is given by

2 $$\begin{array}{c}\langle m\rangle =\eta \langle n\rangle \\ \langle {m}^{2}\rangle ={\eta }^{2}\langle {n}^{2}\rangle +\eta \,\mathrm{(1}-\eta )\langle n\rangle \mathrm{.}\end{array}$$

Hence, the analogous of ${g}_{n}^{\mathrm{(2)}}$ for detected photons, which is defined as ${g}^{\mathrm{(2)}}=\langle {m}^{2}\rangle /{\langle m\rangle }^{2}$, can be easily connected to ${g}_{n}^{\mathrm{(2)}}$ by^23^:

3 $${g}^{\mathrm{(2)}}=\frac{{\eta }^{2}\langle {n}^{2}\rangle +\eta \,\mathrm{(1}-\eta )\langle n\rangle }{{\eta }^{2}{\langle n\rangle }^{2}}={g}_{n}^{\mathrm{(2)}}+\mathrm{1/}\langle m\rangle \mathrm{.}$$

This expression yields

4 $${g}_{n}^{\mathrm{(2)}}-1=(F-\mathrm{1)/}\langle m\rangle ,$$

where $F={\sigma }^{2}(m)/\langle m\rangle$ is the Fano factor for detected photons and ${{\rm{\sigma }}}^{2}(m)$ is the variance. This relation shows that a value of Fano factor lower than 1 implies the antibunching effect and viceversa. Satisfying Eq. (4) can be challenging for bright beams and in the presence of a low quantum efficiency of the detection apparatus. In fact, it can be easily demonstrated that for a detector with quantum efficiency *η*, the Fano factor for detected photons, *F*, is related to that of photons, *F*_*n*_ as

5 $$F=\eta {F}_{n}+\mathrm{(1}-\eta \mathrm{).}$$

For small values of *η*, the detected Fano factor always becomes close to unity. In these critical situations, a powerful method to investigate the statistical properties of light is given by the calculation of the shot-by-shot photon-number correlations between the two outputs of a BS with transmittance *τ* at which the light under study has been divided^10,23^. As a matter of fact, correlations can be exploited to highlight the fluctuations of photon numbers, beyond the direct reconstruction of the photon-number distribution. For instance, in a recent work we have measured correlations to emphasize the difference between a thermal statistics and a super-thermal one^24^.

We define the correlation coefficient for detected photons as

6 $${\rm{\Gamma }}=\frac{\langle ({m}_{c}-\langle {m}_{c}\rangle )({m}_{d}-\langle {m}_{d}\rangle )\rangle }{\sqrt{{\sigma }^{2}({m}_{c}){\sigma }^{2}({m}_{d})}},$$

where *c* and *d* identify the two BS outputs. The coefficient Γ can be also expressed in terms of the first two moments of the input state, or, in a more compact form, in terms of its Fano Factor:

7 $${\rm{\Gamma }}=\frac{\sqrt{\tau ^{\prime} \tau ^{\prime\prime} }(F-\mathrm{1)}}{\sqrt{[F\tau ^{\prime} +\tau ^{\prime\prime} ][F\tau ^{\prime\prime} +\tau ^{\prime} ]}}\mathrm{.}$$

where $\tau ^{\prime} =\tau /{\eta }_{c}$ and $\tau ^{\prime\prime} =\mathrm{(1}-\tau )/{\eta }_{d}$, *η*_c_ and *η*_*d*_ being the quantum efficiencies of the detectors at the two BS outputs. For the sake of simplicity, in the following we will assume ${\eta }_{c}={\eta }_{d}$. Note that in this case the expression of Γ for detected photons is identical to that for photons^25^. From Eq. (7) it is clear that when a super-Poissonian state of light (for which *F* > 1) is divided at a balanced BS, positive values of Γ are expected. A particular case is that of a multi-mode thermal state, for which $F=(\langle m\rangle /\mu +1)\,\mathrm{ > }\,1$ and hence Γ > 0: if the number of modes *μ* is large with respect to the mean-photon number 〈*m*〉, Γ approaches 0. On the contrary, sub-Poissonian states of light, for which *F* < 1, give rise to negative values of Γ.

As already described^21,26^, performing conditional operations on a multi-mode TWB allows the generation of sub-Poissonian states whose Fano factor reads as follows^21^ (see Sect. “Methods - Conditional measurements on multi-mode twin beam states”):

8 $$F=\mathrm{(1}-\eta )[\frac{\langle m\rangle ({m}_{{\rm{cond}}}+\mu )(\langle m\rangle +\eta \mu )}{(\langle m\rangle +\mu )[({m}_{{\rm{cond}}}+\mu )(\langle m\rangle +\eta \mu )-\eta \mu (\langle m\rangle +\mu )]}+1],$$

where *η* is the overall detection efficiency, 〈*m*〉 the mean value of the unconditioned state, and *m*_cond_ the conditioning value, that is the value of photons measured in the idler according to which the values of the signal are selected. The expression in Eq. (8) shows a complex relationship of the involved parameters, which consequently affects the correlation coefficient Γ in Eq. (7).

In the scheme we consider in this paper, the existence of nonclassical correlations between the signal (or a portion of it) and the idler of a TWB state is a necessary condition for the generation of sub-Poissonian states of light by means of conditioning operations performed in post processing and for the observation of anti-correlations between the conditional states. The use of the BS at which the signal is divided offers the possibility to perform this operation on two beams at the same time. In such a way, two nonclassical states can be simultaneously generated. However, it is important to remark that the resulting states are endowed with a lower level of nonclassicality due to the non-unit transmittance of the BS.

### Experimental implementation

According to the setup extensively described in Sect. “Methods - Experimental setup”, we sent the fourth harmonic pulses of a mode-locked Nd:YLF laser to a BBO crystal to produce PDC in a quasi-collinear interaction geometry. The generated TWB states are intrinsically multi-mode and thus both signal and idler are described by a multi-mode thermal statistics.

**Figure 1:**
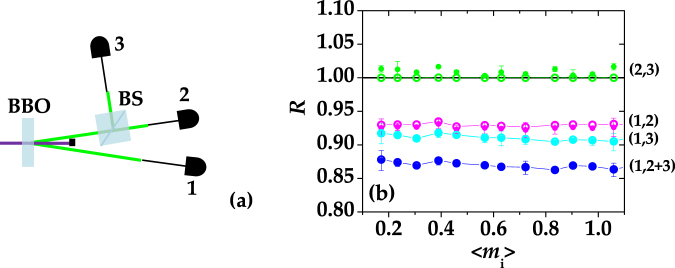
(**a**) Sketch of the experimental scheme. (**b**) *R* as a function of $\langle {m}_{{\rm{i}}}\rangle$ calculated between the idler and each output of the BS (magenta and cyan dots), the idler and the sum of the two BS outputs (blue dots), and the two BS outputs (green dots). The corresponding theoretical expectations are shown as empty circles + lines (for guiding the eye) with the same choice of colors. The black solid line defines the shot-noise level.

A TWB chosen around frequency degeneracy was spatially and spectrally filtered. The portion of the idler was directly measured by a hybrid photodetector (HPD), whereas that of the signal was divided at a balanced BS before being detected by a pair of HPDs collecting the light at the BS outputs (see Fig. 1(a) for details). By applying the self-consistent procedure described in Sect. “Methods - Analysis of the signal”, we could convert the output voltages of the detection chains into numbers of detected photons. The values of the detected photons  were measured shot-by shot and the statistics and correlations of detected photons were evaluated. In particular, the statistics was expected to be a multi-mode thermal, and from its mean value, 〈*m*_*k*_〉, and variance, ${\sigma }^{2}({m}_{k})$ ($k=1,2,3$), it was possible to determine the number of modes as ${\mu }_{k}={\langle {m}_{k}\rangle }^{2}/({\sigma }^{2}({m}_{k})-\langle {m}_{k}\rangle )$ ^27^. Note that all the quantities presented hereafter are expressed in terms of detected photons.

To establish the nature of the photon-number correlations between all the pairs of fields (see the labels in Fig. 1), we considered the noise reduction factor *R*, defined as the variance of the photon-number difference normalized to the shot-noise level

9 $$R=\frac{{\sigma }^{2}({m}_{j}-{m}_{k})}{\langle {m}_{j}+{m}_{k}\rangle }.$$

It is possible to demonstrate that in the case of a perfect multi-mode twin-beam state, the noise reduction factor in Eq. (9) can be written as

10 $$R=1-\frac{2{\eta }_{1}{\eta }_{2}}{{\eta }_{1}+{\eta }_{2}}+\frac{{({\eta }_{1}-{\eta }_{2})}^{2}}{{\eta }_{1}+{\eta }_{2}}\frac{\langle n\rangle }{\mu },$$

where *η*_1_ and *η*_2_ are the quantum efficiencies of the detection chains in the two arms under consideration. When ${\eta }_{1}={\eta }_{2}=\eta$ the expression reduces to $R=1-\eta$, which is the minimum value that can be achieved with detected photons. Note also that values of *R* between 1 − *η* and 1 are sufficient conditions for entanglement^28,29^. On the contrary, *R* ≥ 1 testifies the presence of classical correlations. Since in the experiment the mean number of photons per mode is quite low (we measured $\langle {m}_{k}\rangle /{\mu }_{k} < 0.01$ in each detection chain), for each combination of fields the expression in Eq. (10) reduces to $R=1-\mathrm{(2}{\eta }_{j}{\eta }_{k})/({\eta }_{j}+{\eta }_{k})$, in which $\mathrm{(2}{\eta }_{j}{\eta }_{k})/({\eta }_{j}+{\eta }_{k})=\tilde{\eta }$ is the harmonic mean. With such an assumption and considering the experimental values of *R*, Eq. (10) can be used to obtain the value of $\tilde{\eta }$.

In Fig. 1(b), we show the experimental values of *R* obtained by considering all the possible combinations of fields, namely the two outputs, “2” and “3”, of the BS at which the signal has been divided (green dots), the idler, “1”, and the portion of signal transmitted, “2”, (magenta dots)/reflected, “3”, (cyan dots) by the BS, and finally the idler, “1”, and the sum of the two outputs, “2 + 3”, (blue dots). All of them are plotted as functions of the mean number of photons detected in the idler arm, 〈*m*_*i*_〉. The only case in which we have classical correlations, that is non-sub-shot-noise correlations, is between the two BS outputs. In fact, the signal statistics is super-Poissonian and its division at a BS can only give rise to classical correlations (see Eq. (7))^10^. In the other three cases, we have sub-shot-noise correlations. It is worth noting that if one neglects possible losses due to the presence of the BS, the noise reduction factor calculated between the idler and the sum of the BS outputs is a good approximation of the correlations between the whole signal and idler. The overall efficiency evaluated with this assumption is 0.14.

In Fig. 1(b), together with the experimental data we also plot the theoretical expectations (empty circles + lines to guide the eye) of *R* valid in the case of a multi-mode thermal  TWB^30^:

11 $$R=1-2\tilde{\eta }\frac{\sqrt{\langle {m}_{j}\rangle \langle {m}_{k}\rangle }}{\langle {m}_{j}\rangle +\langle {m}_{k}\rangle }+\frac{{(\langle {m}_{j}\rangle -\langle {m}_{k}\rangle )}^{2}}{\mu (\langle {m}_{j}\rangle +\langle {m}_{k}\rangle )}\mathrm{.}$$

We note that the theoretical values of *R* shown in Fig. 1(b) were calculated by inserting in Eq. (11) the measured values of the mean numbers of detected photons 〈*m*_*j*_〉 and 〈*m*_*k*_〉, of the quantum efficiency $\mathop{\eta }\limits^{ \sim }$, and of the number of modes *μ* ^21,30^. In particular, the values of 〈*m*_*j*_〉 and 〈*m*_*k*_〉 were obtained by the reconstruction of the statistics of detected photons, $\tilde{\eta }$ was obtained as $\tilde{\eta }=1-R$, in which we used the measured values of *R*, and *μ* was derived from the first two moments of the statistics of detected photons.

**Figure 2:**
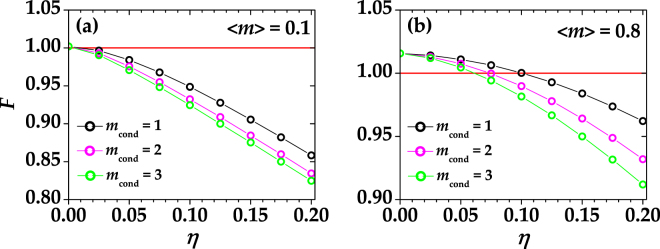
Theoretical values of *F* as functions of *η* for different choices of the conditioning value, *m*_cond_. Black dots + line: *m*_cond_ = 1; magenta dots + line: *m*_cond_ = 2; green dots + line: *m*_cond_ = 3. The red solid line defines the condition *F* = 1. In both panels we fixed the mean number of photons (〈*m*〉 = 0.1 in panel (a) and 〈*m*〉 = 0.8 in panel (b)) and the number of modes (*μ* = 50 in both panels).

As anticipated in Sect. “Photon-number correlations”, the nonclassical correlations in the TWB allow the generation of sub-Poissonian states of light by means of conditional measurements. This is also true if we consider a conditional operation between the idler and each of the BS outputs. Following the procedure described in Sect. “Methods”, according to the number of photons detected in the idler, we post-selected the corresponding data samples measured at each BS output. For each choice of conditioning value, the photon-number distribution was reconstructed, and the Fano factor evaluated. According to Eq. (8), a Fano factor lower than 1 is actually achievable if the global quantum efficiency of the detection system is not too low. Indeed, the presence of losses can be detrimental for the observation of a nonclassical behavior since they act as a reduced quantum efficiency. For the sake of clarity, in Fig. 2 we show the expected values of *F* as a function of *η* at different conditioning values for fixed choices of the number of modes and of the mean number of photons in the unconditioned state. In particular, we notice that the lower the value of 〈*m*〉 the easier the observation of the sub-Poissonian character of the conditional state.

Since the presence of the 50% BS in our setup can be viewed as a loss for the detectors on arms 2 and 3, the estimation of the quantum efficiency for the cases (1,2) and (1,3) is ~0.07 compared to 0.14 for (1,2 + 3) (see Fig. 1(b)).

**Figure 3:**
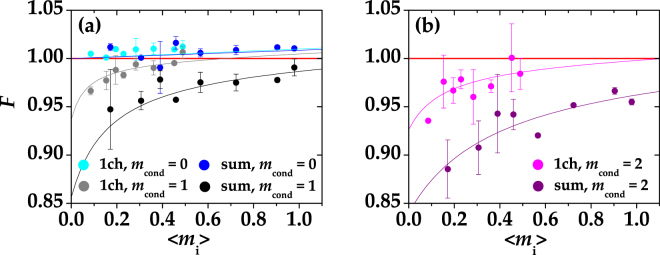
Fano factor of the conditional states obtained at one BS output and by summing the two outputs as a function of $\langle {m}_{{\rm{i}}}\rangle \mathrm{/2}$ and of 〈*m*_i_〉, respectively, for different choices of *m*_cond_. Panel (a): *F* for *m*_cond_ = 0 (cyan: single BS output, blue: sum of the two BS outputs) and *m*_cond_ = 1 (grey: single BS output, black: sum of the two BS outputs). Panel (b): *F* for *m*_cond_ = 2 (magenta: single BS output, purple: sum of the two BS outputs). In each panel, the theoretical expectations (see the text for details) are shown as lines with the same choice of colors. The red solid line defines the condition *F* = 1.

In Fig. 3 we present the Fano factor of the conditional states obtained from the experimental data for different conditioning values *m*_cond_. In particular, the Fano factor of the conditional states obtained at one BS output (indicated as “1ch” in the legend) is shown as a function of $\langle {m}_{{\rm{i}}}\rangle \mathrm{/2}$, while the Fano factor of the states obtained by conditioning the sum of the two outputs (indicated as “sum” in the legend) is shown as a function of 〈*m*_i_〉 in order to be displayed simultaneously.

We note that the condition *m*_cond_ = 0 (cyan and blue symbols in panel (a)) corresponds to a Gaussian operation that does not change the original nature of the conditional states. At variance with this case, choosing *m*_cond_ = 1 (grey and black symbols in panel (a)) and *m*_cond_ = 2 (magenta and purple symbols in panel (b)) leads to the generation of sub-Poissonian states. It is worth noting that the lowest values of *F* are attained in the cases in which the conditioning procedure was applied to the sum of the two BS outputs. This fact testifies the crucial role played by losses in the generation of sub-Poissonian conditional states.

The full lines in Fig. 3 are the theoretical fit functions calculated according to Eq. (8) in which we set *μ* = 100 (that roughly corresponds to the mean number of modes measured in each arm) and *η* is the only fitting parameter. We note that the values of *η* obtained from the fit are compatible with those evaluated from Fig. 1(b).

**Figure 4:**
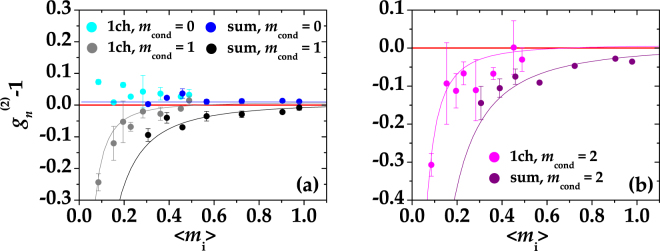
Values of *g*_*n*_^(2)^−1 for the conditional states obtained at one BS output and by summing the two outputs plotted as functions of $\langle {m}_{{\rm{i}}}\rangle \mathrm{/2}$ and of 〈*m*_i_〉, respectively, for different choices of *m*_cond_. Panel (a): *g*_*n*_^(2)^−1 for *m*_cond_ = 0 (cyan: single BS output, blue: sum of the two BS outputs) and *m*_cond_ = 1 (grey: single BS output, black: sum of the two BS outputs). Panel (b): *F* for *m*_cond_ = 2 (magenta: single BS output, purple: sum of the two BS outputs). The corresponding theoretical expectations (see the text for details) are shown as lines with the same choice of colors. The red solid line defines the condition *g*_*n*_^(2)^ = 1.

For the sake of completeness, in Fig. 4 we plot the quantity ${g}_{n}^{\mathrm{(2)}}-1={g}^{\mathrm{(2)}}-\mathrm{1/}\langle m\rangle -1$, corresponding to the autocorrelation function, subtracted of the value 1, of the states obtained by conditioning either one BS output (indicated as “1ch” in the legend) or the sum of the two BS outputs (indicated as “sum” in the legend) as a function of $\langle {m}_{{\rm{i}}}\rangle \mathrm{/2}$ and 〈*m*_i_〉, respectively. Indeed, as already anticipated in Sect. “Photon-number correlations”, lower-than-1 values of the quantity *g*_*n*_^(2)^−1 imply the presence of antibunching behavior. The conditions *m*_cond_ = 0, 1 are shown in panel (a) and *m*_cond_ = 2 in panel (b) with the same color coding used in Fig. 3. The theoretical curves (full lines) were obtained according to Eq. (4), in which we set *μ* = 100 (that corresponds to the typical number of modes measured in each arm in this experiment), and *η* is the only fitting parameter. Once again the values of *η* obtained from the fits are comparable with those estimated from the other quantities.

If on the one hand the negativity of *g*_*n*_^(2)^−1 is expected, as it is evaluated from the same quantities involved in the definition of the Fano factor (mean value and variance), on the other hand it attests the quality and robustness of our data.

**Figure 5:**
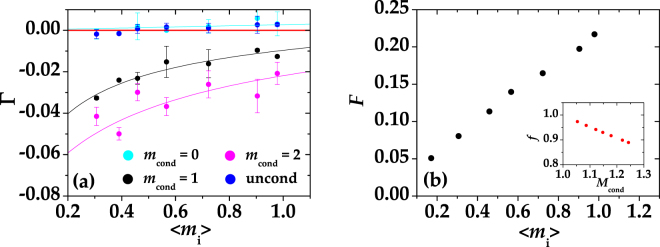
(**a**) Γ as a function of 〈*m*_i_〉 for different choices of the conditioning value *m*_cond_. Blue dots: unconditioned states; cyan dots: *m*_cond_ = 0; black dots: *m*_cond_ = 1; magenta dots: *m*_cond_ = 2. The corresponding theoretical expectations (see the text for details) are shown as lines with the same choice of colors. The red solid line defines the condition Γ = 0. (**b**) Fano factor of the conditional states obtained at one BS output and shown in Fig. 3(a) upon removal of the contribution of the zero peak as a function of 〈*m*_i_〉 for *m*_cond_ = 1. Inset: Fidelity of the conditional states to the Fock state |1〉 as a function of the mean value *M*_cond_ of the conditional states.

As anticipated in the Introduction, in the mesoscopic regime the photon-number correlations between the two BS outputs can be considered as an alternative method to investigate the nonclassicality of the conditional states. In Fig. 5(a) we plot the shot-by-shot photon-number correlations between pairs of conditional states produced in post processing at the BS outputs as functions of $\langle {m}_{{\rm{i}}}\rangle$. First of all, we notice that the correlations between the two unconditioned states at the two BS outputs, shown as blue dots in the figure, are slightly positive. As already noticed, in this case the absolute values of Γ are close to 0 because the number of photons per mode is quite low. On the contrary, the correlations between the conditional states produced at the two BS outputs, shown as, black and magenta dots for increasing values of *m*_cond_ exhibit negative values, thus attesting the antibunching behavior. Moreover, as expected from the theory, the absolute values of the correlation coefficient increase at increasing the conditioning value. Like in the previous figures, superimposed to the experimental data shown as dots, we plot the theoretical expectations according to Eq. (7), in which we set *μ* = 100 (that roughly corresponds to the mean number of modes measured in each arm), *τ* equal to the experimental balancing between the two BS outputs and the only fitting parameter is again the global quantum efficiency *η*, whose value is again compatible with that obtained for the previous figures. As a final remark to Figs 3–5, we note that the presence of some large error bars can be ascribed to the reduced size of the data samples after the application of the different conditioning operations. In particular, the largest error bars correspond to the data obtained by choosing *m*_cond_ = 2. Despite this fact, we notice that all the data shown in Figs 3–5 clearly exhibit a trend, which is well reproduced by the corresponding theoretical expectations.

The data presented in Fig. 3 demonstrate the possibility to preserve the nonclassicality of quantum states despite the losses introduced by the BS. However, it is important to remark that the key role in evidencing the nonclassical character of the states is played by the overall efficiency of the detection apparatus. In our case, the detection efficiency is quite low, thus increasing the number of events in which the detector does not reveal anything. By analogy with the operation of a single-photon detector, for which only the events corresponding to “clicks” contribute to the signal, it would be desirable to remove the contribution of the zero-photon events from the photon distribution. This choice would enhance the sub-Poissonian character of the conditional states.

From the operational point of view, removing the “0” from the photon-number statistics of PNR detectors corresponds to the application of a threshold, below which the signal coming from the detection apparatus is not registered. As shown in Fig. 5(b), in this case the absolute values of *F* corresponding for instance to the conditioning value *m*_cond_ = 1 are very close to zero, thus testifying the good quality of the generated sub-Poissonian states. To quantify the results of such an operation, we compare the photon-number distribution of the resulting states to that of the Fock state |1〉. In particular, in the Inset of Fig. 5(b) we show the fidelity $f={\sum }_{m}\sqrt{{p}_{m}^{{\rm{th}}}{p}_{m}^{\exp }}$, where ${p}_{m}^{{\rm{th}}}$(${p}_{m}^{\exp }$) is the theoretical (experimental) distribution and the sum extends up to the maximum number of detected photons $\bar{m}$ above which both the distributions become negligible, as a function of the mean value *M*_cond_ of the conditional states. We notice that values of fidelity as high as 0.97 are achievable.

## Discussion

In conclusion, we have realized a compact setup involving a balanced BS and three PNR detectors for the observation of photon antibunching at a mesoscopic intensity level. This method allows us to better characterize the statistical properties of sub-Poissonian states obtained in post processing by conditional measurements. The generation of sub-Poissonian light by conditional operations on TWB states is a well-known result^21^. By adding a BS, the present scheme demonstrates that the nonclassical character of the conditional states can survive losses so that the BS generates a pair of sub-Poissonian states. Moreover, the two states are anti-correlated, as expected from a sub-Poissonian light passing through a BS. Finally, by setting a proper threshold on the output of the detectors, so that only the events containing at least one detected photon are recorded, a good approximation of a single-photon Fock state has been obtained.

In view of further improvements and applications, such results suggest the implementation of a cascading configuration including additional BSs for the simultaneous generation of multiple copies of sub-Poissonian states. Moreover, this investigation based on the use of a balanced BS encourages the detection of nonclassical states by means of a homodyne-like detection scheme with PNR detectors, which we recently realized and used to deal with the problem of discrimination among coherent states in the presence of phase noise^31^.

## Methods

### The mesoscopic intensity domain

The mesoscopic intensity domain, in which we performed our work, is the regime in which pulsed optical states of light containing sizeable numbers of photons are produced, measured and exploited. To accomplish the crucial issue of light detection in this domain, in the last two decades different classes of PNR detectors have been produced and exploited to characterize quantum states of light^32^. Among them, it is worth mentioning the HPD used in the present work, which combines a photocathode with a diode structure operated below the breakdown threshold^11,30^ as the amplification stage. At variance with the traditional photomultipliers, in such a detector the amplification process occurs in a single step, so that the excess noise is small enough to allow photon-number resolution (up to 6 detected photons). As an alternative, the accomplishment of photon-counting capability can be obtained by splitting the light to be measured either in space or in time prior to detection so that at most one photon at a time hits the detector sensitive area. Among these detectors, we mention the visible light photon counter (VLPC)^13^; the fiber-loop detector, which is a time-multiplexed detector based on one or more single-photon avalanche diodes (SPADs)^14,15^; the Silicon photomultiplier (SiPM), that is constituted by a matrix of SPADs with a common output. Due to their composite structure, SiPMs have a good photon-counting capability, even if their large dark-count rate (100 kHz in the new generation) and their not negligible cross-talk probability have till now almost prevented their use in the field of Quantum Optics^16–18^. In the last decade, a tremendous progress has been achieved in the field of superconductors, so that new types of detectors have been developed, such as the transition-edge sensors (TES)^19,22^ and the superconducting nanowires^20^. Despite having a good quantum efficiency, these detectors must operate at cryogenic temperatures and thus their operation is rather cumbersome. As of today, the ideal detector has yet to appear and the optimal choice is application specific.

### Experimental setup

**Figure 6:**
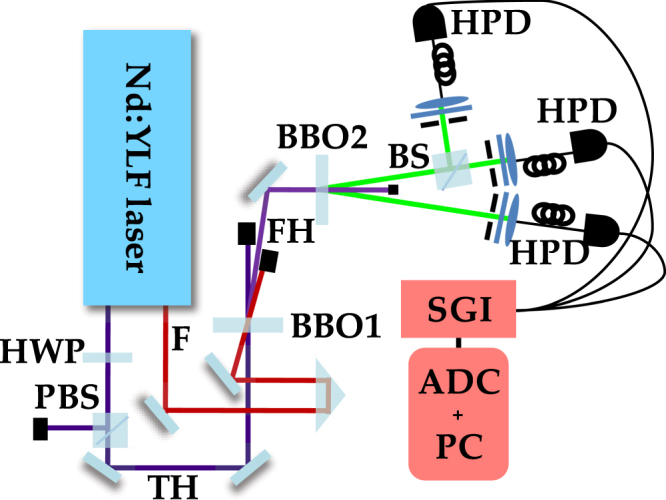
Sketch of the experimental scheme. F: Fundamental beam; TH: Third-harmonic beam; FH: Fourth-harmonic beam; BBO1: *β*-barium borate crystal used for the generation of the fourth-harmonic beam; BBO2: *β*-barium borate crystal used for the generation of the PDC process; HWP: Half-wave plate; PBS: Polarizing cube beam splitter; BS: balanced beam splitter; HPD: Hybrid photodetector; SGI: syncronous-gated integrator; ADC + PC: analog-to-digital converter + acquisition system.

The experimental implementation of our scheme has been obtained by means of the setup sketched in Fig. 6. The fourth harmonics (FH in the figure) at 262.2 nm of a mode-locked Nd:YLF laser regeneratively amplified at 500 Hz was obtained by mixing the fundamental beam (F in the figure) at 1047 nm and the third-harmonics (TH) at 349 nm in a BBO crystal (cut angle = 37 deg, 8-mm long) by means of a non-collinear interaction geometry. The fourth hamonics was then sent to a BBO crystal (cut angle = 46.7 deg, 6-mm long) to produce PDC in a quasi-collinear interaction geometry.

Two twin portions around frequency degeneracy (523 nm) were spatially and spectrally filtered by means of two variable irises and bandpass filters. The light on the idler arm was focused by means of an achromatic doublet into a 600-*μ*m-core multi-mode fiber and delivered to a PNR detector. The light on the signal was mixed with the vacuum at a balanced BS. At the two outputs, two collimators were used to collect the light and send it to a pair of PNR detectors through two 600-μm-core multi-mode fibers. As the PNR detectors we used three hybrid photodetectors (HPD, mod. R10467U-40, Hamamatsu, nominal quantum efficiency ~50% at 523 nm), whose outputs were amplified (preamplifier A250 plus amplifier A275, Amptek), synchronously integrated over a 500-ns window (SGI, SR250, Stanford), and digitized (ADC + PC, PCI-6251, National Instruments)^11^. We acquired sequences of 3 × 10^5^ shots at different mean values of the fourth harmonics, whose energy was changed by means of a half-wave plate (HWP) followed by a polarizing cube BS (PBS).

### Analysis of the signals

As mentioned above, The HPDs are commercial PNR detectors composed of a photocathode, whose maximum quantum efficiency is about 50% at 500 nm, followed by an avalanche diode operated below the breakdown threshold. This detector has the advantage of operating at room temperature (in our case its temperature is stabilized at 16 °C by means of a water chiller) and can be operated within the visible spectral range. The internal amplification has a gain profile narrow enough to allow photon-number resolution. According to the model developed in previous works^11,12^, the detection process consists of two steps: photodetection by the photocathode and amplification. The first process is described by a Bernoullian convolution, whereas the second one by a very precise factor, which is taken as constant. The value of the gain, *γ*, can be obtained by means of a self-consistent method based on the very light to be measured^11,30^. Note that this value also coincides with the distance between two consecutive valleys in the pulse-heigth spectrum of the detector output. Once the value of *γ* has been determined, the shot-by-shot number of detected photons is obtained by the following procedure: Each output voltage is subtracted of the mean value of the electronic noise measured in the absence of light, then the resulting values are divided by the value of *γ*, determined in one of the two ways described above, and rebinned in unitary bins. It is worth noting that in the application of the method of analysis to HPDs, there is no need to operate background corrections thanks to the fact that the dark-count and after-pulse contributions are negligible in this class of detectors at the present operating regime.

### Conditional measurements on TWB states

The TWB states generated by PDC are intrinsically multi-mode^30^ and can be written as

12 $$|\psi \rangle =\underset{j\mathrm{=1}}{\overset{\mu }{\otimes }}|\psi {\rangle }_{j}\,,$$

where $|\psi {\rangle }_{j}$ is the single-mode TWB state

13 $$|\psi {\rangle }_{j}=\sqrt{1-|{\lambda }_{j}{|}^{2}}\sum _{n}{\lambda }_{j}^{n}|n\rangle |n\rangle ,$$

|*n*〉 being the state containing *n* photons, $${\varphi }_{0}$$ being the phase of the pump field, and $$\lambda \equiv \,\tanh ({g}_{j})\exp (i{\varphi }_{0})$$, bein*g g*_*j*_ the PDC gain. The mean photon-number value in the generated signal and idler of the state in Eq. (13) is $${\langle n\rangle }_{j}={\rm{s}}{\rm{i}}{\rm{n}}{{\rm{h}}}^{2}({g}_{j})$$, whereas the photon-number statistics is a thermal distribution

14 $${p}_{n,j}=\frac{{\langle n\rangle }_{j}^{n}}{{(1+{\langle n\rangle }_{j})}^{n+1}},$$

so that the TWB state can be rewritten as

15 $$|\psi {\rangle }_{j}=\sum _{n}\sqrt{{p}_{n,j}}\exp (i{\varphi }_{0})|n\rangle |n\rangle \mathrm{.}$$

We note that the single-mode TWB displays perfect photon-number correlations and that the wavefunction is written as a non factorable superposition of number states, meaning that the TWB state is entangled.

Assuming that the *μ* modes in Eq. (12) are equally populated, the mean photon number of all *j* contributions is the same. Hence the mean total number of photons in the multi-mode state is $\langle n\rangle =\mu {\langle n\rangle }_{j}$ and the statistics of photons in each arm of the TWB is given by the multi-mode thermal distribution

16 $${p}_{n}=\frac{(n+\mu -1)!}{n!(\mu -1)!{(\langle n\rangle /\mu +1)}^{\mu }{(\mu /\langle n\rangle +1)}^{n}}\,\mathrm{.}$$

We can thus write the multi-mode TWB in the following compact form

17 $$|\psi \rangle =\sum _{n\mathrm{=0}}^{\infty }\sqrt{{p}_{n}}|{n}^{\otimes }\rangle |{n}^{\otimes }\rangle ,$$

where $|{n}^{\otimes }\rangle =\delta (n-{\sum }_{h\mathrm{=1}}^{\mu }{n}_{h})\,{\otimes }_{k\mathrm{=1}}^{\mu }|n{\rangle }_{k}$ represents the overall *n* photons coming from the *μ* modes that impinge on the detector and *p*_*n*_ is the multi-mode thermal distribution in Eq. (16). Note that also for the multi-mode TWB, perfect correlations in the number of photons are expected.

Equations (14) and (16) describe the statistical distributions for the number of photons in the states as they would be detected by a perfect detector. This is obviously not the case for real photodetectors, which reveal light with a non-unit detection efficiency. If the detection process is described by a Bernoullian convolution, the functional form of Eq. (16) remains the same, only changing $\langle n\rangle \to \eta \langle n\rangle \equiv \langle m\rangle$. The joint probability distribution of detected photons is given by^26^:

$$P({m}_{{\rm{s}}},{m}_{{\rm{i}}})={(\frac{\mu \eta }{\langle m\rangle +\mu \eta })}^{\mu }{(\frac{\eta }{1-\eta })}^{{m}_{{\rm{s}}}+{m}_{{\rm{i}}}}\times \sum _{{\rm{l}}=\,{\rm{\max }}({m}_{{\rm{s}}},{m}_{{\rm{i}}})}^{\infty }[\frac{\langle m\rangle {\mathrm{(1}-\eta )}^{2}}{\langle m\rangle +\mu \eta }](\begin{array}{c}l+\mu -1\\ l\end{array})\,(\begin{array}{c}l\\ {m}_{{\rm{s}}}\end{array})\,(\begin{array}{c}l\\ {m}_{{\rm{i}}}\end{array}),$$

where symbols s and i refer to signal and idler, respectively, and $\langle m\rangle$ is the mean number of photons detected on each of the two beams.

By exploiting the nonclassical photon-number correlations between signal and idler it is possible to implement a conditional procedure that produces a conditional state having completely different statistics. In fact, the conditioning procedure is highly nonlinear, and may produce new quantum states that cannot be generated by other processes.

In the present case, the conditioning procedure we implemented consists in deciding a specific value, say *m*_cond_, for the number of detected photons in the single shots in the idler arm and in selecting the corresponding values in the signal arm. The resulting photon-number distribution is thus a conditional statistics $P({m}_{{\rm{s}}}|{m}_{{\rm{i}}}={m}_{{\rm{cond}}})$, described by the Fano factor in Eq. (8). Note that for *m*_cond_ = 0, *F* > 1, while it can be less than one for appropriate values of *η* and 〈*m*〉 (see Fig. 3).
